# circRNA_104075 stimulates YAP-dependent tumorigenesis through the regulation of HNF4a and may serve as a diagnostic marker in hepatocellular carcinoma

**DOI:** 10.1038/s41419-018-1132-6

**Published:** 2018-10-25

**Authors:** Xiao Zhang, Yanfeng Xu, Zijun Qian, Weisheng Zheng, Qi Wu, Yan Chen, Guoqing Zhu, Ya Liu, Zhixuan Bian, Wen Xu, Yue Zhang, Fenyong Sun, Qiuhui Pan, Jiayi Wang, Lutao Du, Yongchun Yu

**Affiliations:** 1 Department of Clinical Laboratory Medicine, Shanghai Tenth People’s Hospital of Tongji University, 200072 Shanghai, China; 2 Department of Pharmacy, Shanghai Municipal Hospital of Traditional Chinese Medicine, Shanghai University of Traditional Chinese Medicine, 200071 Shanghai, China; 3 Shanghai Municipal Hospital of Traditional Chinese Medicine, Shanghai University of Traditional Chinese Medicine, 200071 Shanghai, China; 4 School of Life Science and Technology, Shanghai Key Laboratory of Signaling and Disease Research, Tongji University, 200092 Shanghai, China; 5 Department of Laboratory Medicine, Shanghai Children’s Medical Center, Shanghai Jiaotong University School of Medicine, 200127 Shanghai, China; 6 Department of Laboratory Medicine, Zhongshan Hospital, Fudan University, 200025 Shanghai, China; 7 Department of Central Laboratory, Shanghai Tenth People’s Hospital of Tongji University, 200072 Shanghai, China; 8 Department of Clinical Laboratory, The Second Hospital of Shandong University, 250033 Jinan, Shandong China; 9 Shanghai Chest Hospital, Shanghai Jiao Tong University, 200030 Shanghai, China

## Abstract

Some types of circular RNA (circRNA) are aberrantly expressed in human diseases including hepatocellular carcinoma (HCC). However, its regulation mechanism and diagnostic roles are largely unknown. Here, we identified that circRNA_104075 (circ_104075) was highly expressed in HCC tissues, cell lines and serum. Mechanistically, HNF4a bound to the −1409 to −1401 region of the circ_104075 promoter to stimulate the expression of circ_104075. Moreover, circ_104075 acted as a ceRNA to upregulate YAP expression by absorbing miR-582-3p. Interestingly, an N^6^-methyladenosine (m^6^A) motif was identified in the 353–357 region of YAP 3′UTR, and this m^6^A modification was essential for the interaction between miR-582-3p and YAP 3′UTR. Further, the diagnostic performance of circ_104075 was evaluated. The area under the receiver operating characteristic (AUC-ROC) for circ_104075 was 0.973 with a sensitivity of 96.0% and a specificity of 98.3%. Collectively, we determined that circ_104075 was highly expressed in HCC and elucidated its upstream and downstream regulatory mechanisms. circ_104075 additionally has the potential to serve as a new diagnostic biomarker in HCC. Targeting circ_104075 may provide new strategies in HCC diagnosis and therapy.

## Introduction

Primary liver cancer is the third most common cause of cancer-related death worldwide^1^. Hepatocellular carcinoma (HCC) is the most common type of primary liver cancer. Because of the lack of early diagnostic biomarkers with high specificity and sensitivity, patients with HCC usually fail to receive timely treatment^2^. The classical biomarkers for clinical diagnosis include α-fetoprotein (AFP)^3^, α-fetoprotein-L3 (AFP-L3)^4^, and des-carboxy-prothrombin (DCP)^5^. However, these biomarkers lead to some false-positive and false-negative results in HCC diagnosis. Therefore, novel diagnostic biomarkers for HCC are still urgently needed.

Since most protein-based assays lack the desired accuracy, non-coding RNA-based assays could be considered as alternative diagnostic tools for HCC^6^. Emerging evidences have suggested that non-coding RNAs play a diagnostic role in HCC^6^. Considering long non-coding RNA (lncRNA), urothelial carcinoma associated-1 (UCA1) has been reported as a biomarker for lncRNA-based HCC diagnostic approach. The reported sensitivities are higher than 90% and the specificities are higher than 82% for UCA1^7,8^. Other lncRNA biomarkers such as HULC^9^, DANCR^10^, and linc01225^11^ are reported to possess good sensitivity and specificity in HCC diagnosis. Moreover, certain types of microRNAs are aberrantly expressed in HCC, and they have the ability to distinguish HCC patients from healthy control subjects. Data from meta-analysis showed that miR-21 exhibits a sensitivity of 86.6% and a specificity of 79.5% in HCC diagnosis^12^. Several studies have provided evidences that miR-223 is upregulated and has the potential to become a diagnostic biomarker in HCC^13–15^.

Compared to linear non-coding RNAs, circular RNA (circRNA) is highly stable because of its covalently closed loop structure^16^. Some types of circRNAs are abnormally expressed in the tissues or serum of HCC patients, and they exhibit pro-tumorigenic roles^17^. For instance, circRNA_10720 promotes EMT by absorbing microRNAs that target vimentin to stimulate HCC tumorigenesis both in vitro and in vivo^18^. Another example is circRNA_0016788, which acts as a sponge for miR-486, stimulates the expression of CDK4, and promotes tumor growth in HCC^19^. Because of its critical function in the development of HCC and its relatively stable characteristics, circRNA exhibits the potential to serve as a novel biomarker in HCC diagnosis.

Here, we revealed that circRNA_104075 was highly expressed in HCC cell line and tissues and serum of HCC patients, and the expression of circRNA_104075 was stimulated by HNF4a. Moreover, circRNA_104075 promoted HCC tumorigenesis by absorbing the inhibitor of YAP, miR-582-3p. N^6^-methyladenosine (m^6^A) modification of the motif in the 353–357 region of YAP 3′UTR promoted YAP inhibition via miR-582-3p. Finally, the diagnostic potential of circRNA_104075 was analyzed, and we found that circRNA_104075 was able to predict the occurrence of HCC. The AUC-ROC for circ_104075 was 0.973 with a sensitivity of 96.0% and a specificity of 98.3%.

## Results

### circ_104075 was highly expressed in HCC

**Fig. 1:**
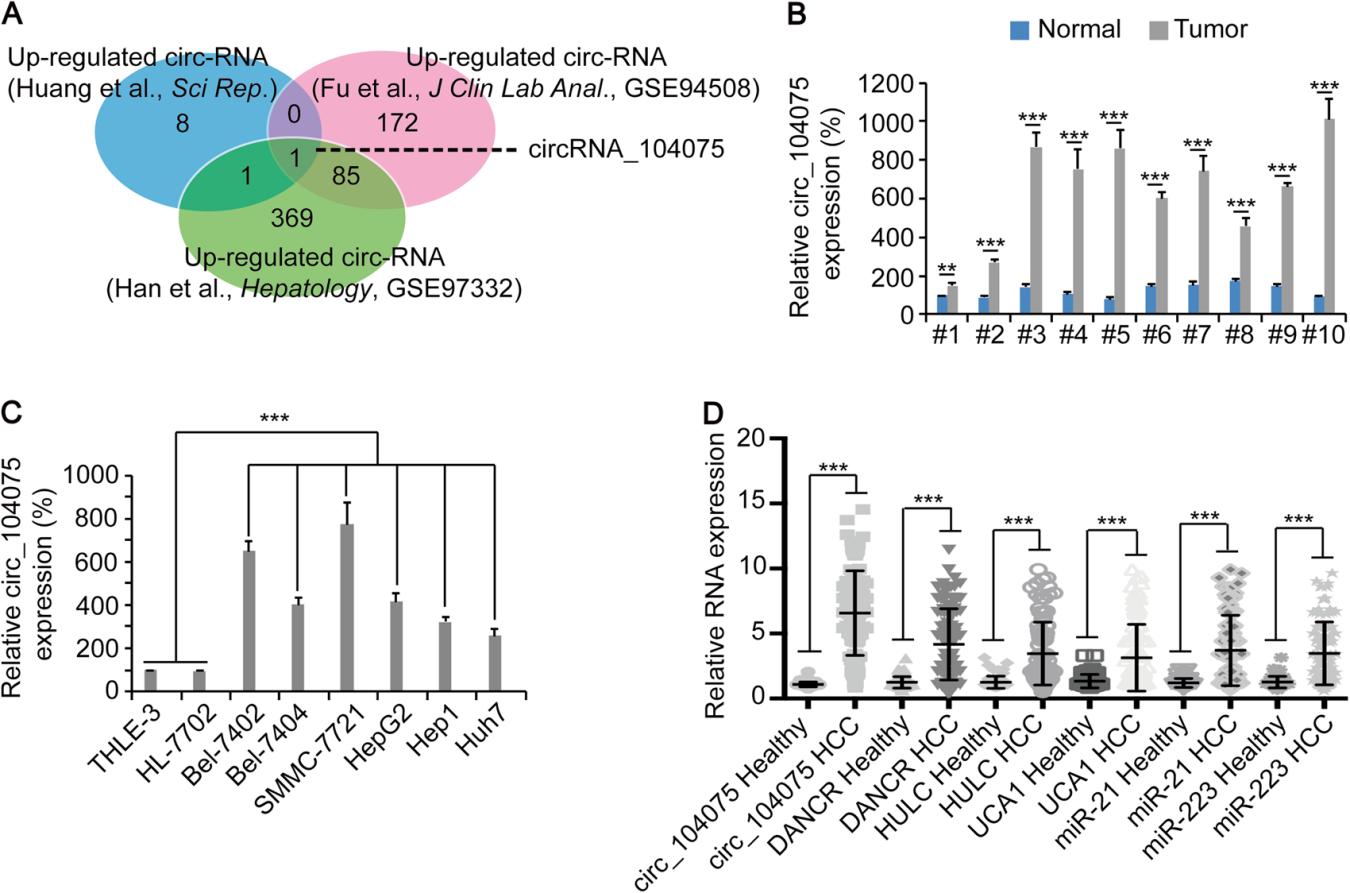
circ_104075 was upregulated in HCC. **a** Venn diagram showing overlapped circRNAs that were highly expressed (log2 FC > 1) in HCC in studies performed by Huang et al., Fu et al., and Han et al., respectively. **b** circ_104075 expression was measured in ten pairs of HCC and adjacent normal tissues using qPCR. **c** circ_104075 expression was measured in normal hepatocytes (THLE-3 and HL-7702) and established HCC cell lines (Bel-7402, Bel-7404, SMMC-7721, HepG2, Hep1 and Huh7) using qPCR. **d** RNA expression of circ_104075, DANCR, HULC, UCA1, miR-21 and miR-223 was measured in the serum of healthy individuals (*n* = 60) and HCC patients (*n* = 101) using qPCR. The data are presented as the means + SD from three biological replicates. **p < 0.01, ***p < 0.001. The data shown in **b**, **c**, and **d** were analyzed using a one-way ANOVA test

Microarray data were collected from three studies on circRNA expression in HCC vs Healthy tissues. Ten circRNAs were identified to be highly expressed in HCC in the study performed by Huang et al.^20^, 258 circRNAs were identified to be highly expressed in HCC in the study performed by Fu et al.^21^, and 456 circRNAs were identified as highly expressed in HCC in the study performed by Han et al.^22^. Only circRNA_104075 (circ_104075) was found to be highly expressed in all three studies (Fig. 1a). Upon evaluating ten pairs of clinical liver tissues, a higher level of circ_104075 was detected in HCC tissues compared to adjacent normal tissues (Fig. 1b). A higher expression of circ_104075 was also observed in established HCC cell lines (Bel-7402, Bel-7404, SMMC-7721, HepG2, Hep1, and Huh7) compared to normal hepatocyte lines (THLE-3 and HL-7702) (Fig. 1c). Moreover, we detected the level of circ_104075 and several reported lncRNA and microRNA HCC biomarkers in the serum. We found that the expression of circ_104075 was much higher in the serum of HCC patients compared to those of healthy individuals, and the average fold-change (circ_104075: 6.03 ± 2.99) was more obvious compared to other lncRNA and microRNA biomarkers (DANCR: 3.34 ± 2.20, HULC: 2.75 ± 1.92, UCA1: 2.34 ± 1.91, miR-21: 3.09 ± 2.26, and miR-223: 2.74 ± 1.90) (Fig. 1d). These results demonstrated that circ_104075 was highly expressed in HCC tissues, cell lines and serum.

### circ_104075 expression was positively regulated by HNF4a in HCC

**Fig. 2:**
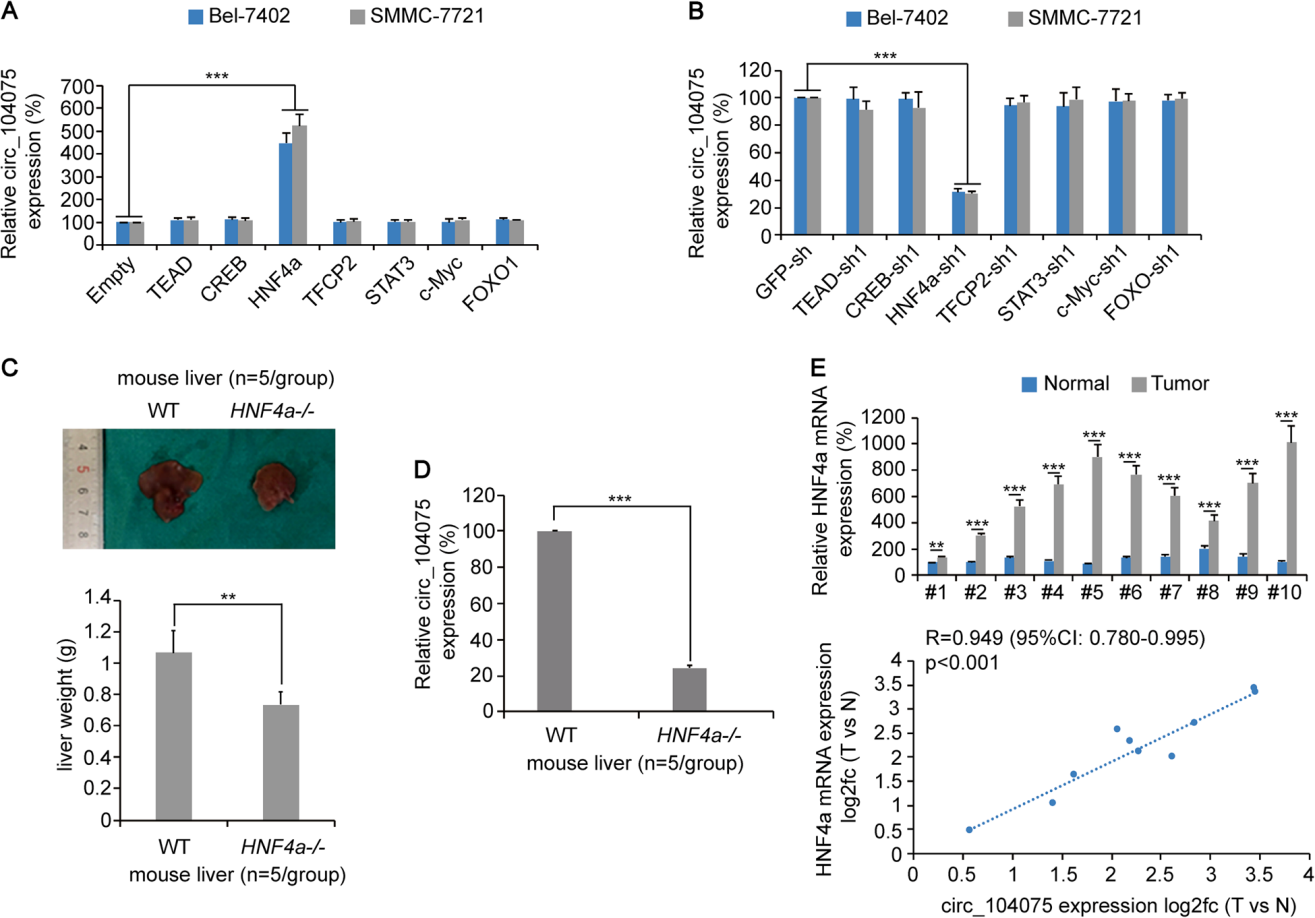
circ_104075 expression was positively regulated by HNF4a. **a** circ_104075 expression was measured using qPCR with empty vectors or vectors of HCC promoting transcription factors transfected in Bel-7402 and SMMC-7721 cells. **b** circ_104075 expression was measured using qPCR with GFP-sh or shRNA targeting HCC promoting transcription factors transfected in Bel-7402 and SMMC-7721 cells. **c** General views of the liver of WT and *HNF4a* knockout *(HNF4a-/-)* mice (upper), and the liver weight was measured (lower), *n* = 5 per group. **d** circ_104075 expression was measured using qPCR in the liver of WT and *HNF4a* knockout *(HNF4a-/-)* mice, n = 5 per group. **e** HNF4a mRNA expression was measured using qPCR in ten pairs of HCC and adjacent normal tissues (upper). Relationship between HNF4a mRNA expression and circ_104075 expression was analyzed (lower). The data are presented as the means + SD from three biological replicates. **p < 0.01, ***p < 0.001. The data shown in **a–d** and (**e**, upper) were analyzed using a one-way ANOVA test. The data shown in **e** (lower) were analyzed using the Spearman rank-correlation analysis. CI, confidence interval

To confirm how circ_104075 expression was stimulated in HCC, we overexpressed or knocked down some well-acknowledged HCC-promoting transcription factors (TEAD^23^, CREB^24^, HNF4a^25^, TFCP2^26^, STAT3^27^, c-Myc^28^, and FOXO1^29^) in the HCC cell lines Bel-7402 and SMMC-7721. We observed that only HNF4a overexpression or knockdown could significantly stimulate or inhibit the expression of circ_104075 (Fig. 2a, b). In the CRISPR/Cas9-generated *HNF4a* knockout (*HNF4a-/-*) mouse models, the size and weight of the liver were lower compared to those of the wild-type mice (Fig. 2c). Moreover, the expression of circ_104075 in the liver of the *HNF4a-/-* mice was lower than that in the wild-type mice (Fig. 2d). We also determined the level of the HNF4a mRNA in clinical liver tissues, and found that the HNF4a mRNA level was higher in the HCC tissues than in adjacent normal tissues. A positive correlation was also identified for the fold-change (HCC vs. normal) between circ_104075 and HNF4a [*R* = 0.949 (95% confidence interval: 0.780–0.995), *p* < 0.001] (Fig. 2e), which suggested that circ_104075 expression might be promoted by HNF4a.

**Fig. 3:**
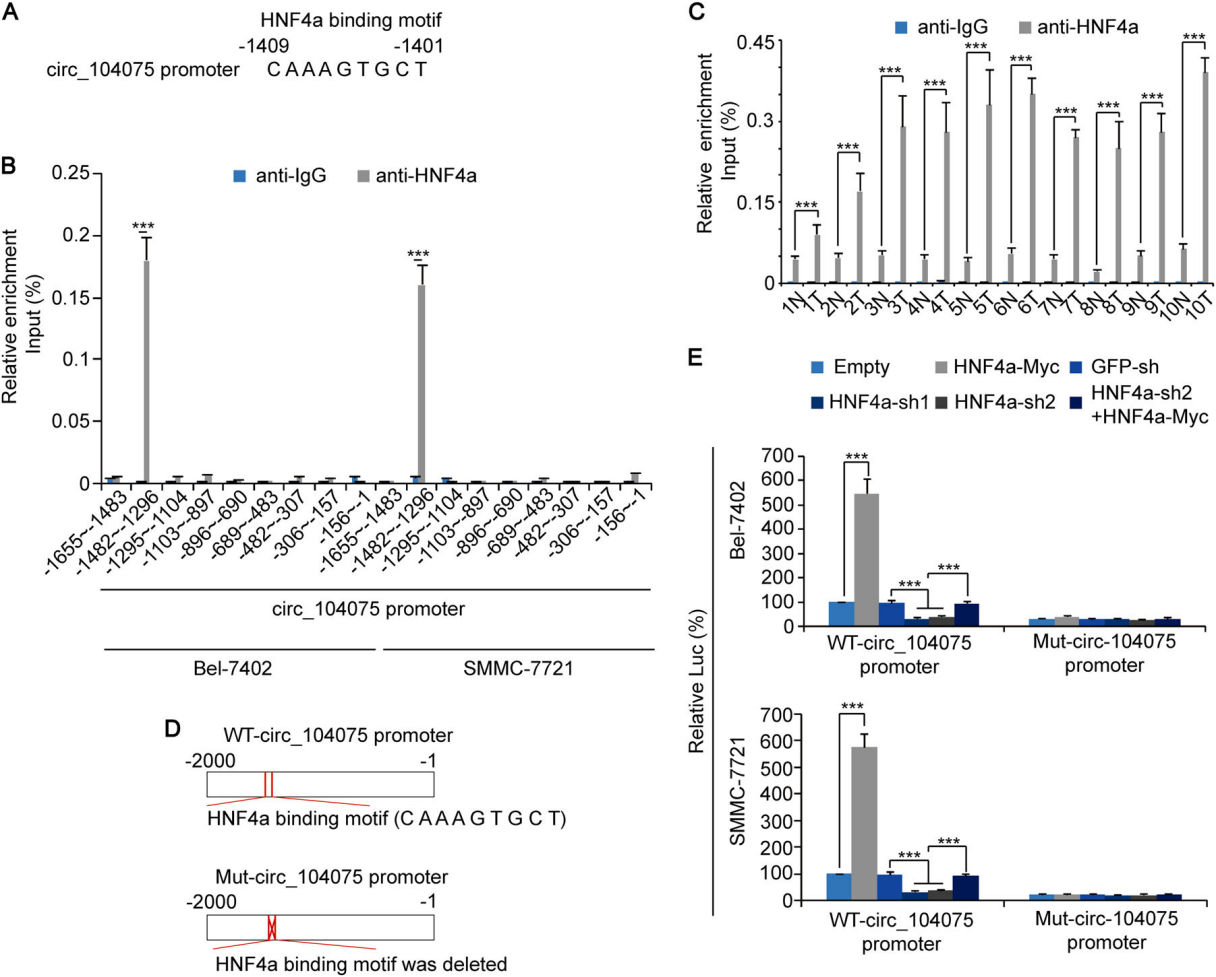
circ_104075 promoter was bound by HNF4a. **a** An HNF4a-binding motif was identified in the −1409 to −1401 region of the circ_104075 promoter using the JASPAR database. **b** The enrichment of HNF4a in Bel-7402 and SMMC-7721 cells at the indicated regions of circ_104075 promoter was calculated as the percentage of the input chromosomal DNA via ChIP using the corresponding antibodies. A non-specific IgG was used as the negative control antibody. **c** The enrichment of HNF4a in ten pairs of HCC and adjacent normal tissues at the −1482 to −1296 region of the circ_104075 promoter was calculated as the percentage of the input chromosomal DNA via ChIP using the corresponding antibodies. A non-specific IgG was used as the negative control antibody. **d** Schematic representation of WT- and Mut-circ_104075 promoter. **e** HNF4a was overexpressed or knocked down using indicated expression plasmids. Activities of WT- or Mut-NRP1-Promoter were measured using the dual-luciferase regent in Bel-7402 (upper) and SMMC-7721 cells (lower). The data are presented as the means + SD from three biological replicates. ***p < 0.001. The data shown in **b**, **c**, **e** were analyzed using a one-way ANOVA test

An HNF4a-binding motif was identified in the −1409 to −1401 region of the circ_104075 promoter (Fig. 3a). ChIP-qPCR results indicated that the region of the circ_104075 promoter (−1482 to −1296) that contained this motif could bind with HNF4a, whereas other regions exhibited no such ability (Fig. 3b). We also performed ChIP-qPCR using the primers designed for the −1482 to −1296 region of the circ_104075 promoter in clinical HCC tissues; the results demonstrated that the circ_104075 promoter (−1482 to −1296 region) was combined with HNF4a in HCC tissues to a greater extent compared to that in the adjacent normal tissues (Fig. 3c). To further confirm the role of the HNF4a-binding motif, luciferase reporters containing WT and mutant (Mut) HNF4a-binding motif circ_104075 promoter were constructed and transfected into Bel-7402 and SMMC-7721 cells, respectively (Fig. 3d). We observed that the luciferase activity of the WT-circ_104075 promoter was stimulated by HNF4a overexpression and was suppressed by HNF4a knockdown. In contrast, when the binding motif of HNF4a was mutated, the luciferase activity was not regulated by HNF4a overexpression or knockdown (Fig. 3e). These data demonstrated that circ_104075 transcription was stimulated by HNF4a.

### circ_104075 stimulated YAP expression via absorbing miR-582-3p

**Fig. 4:**
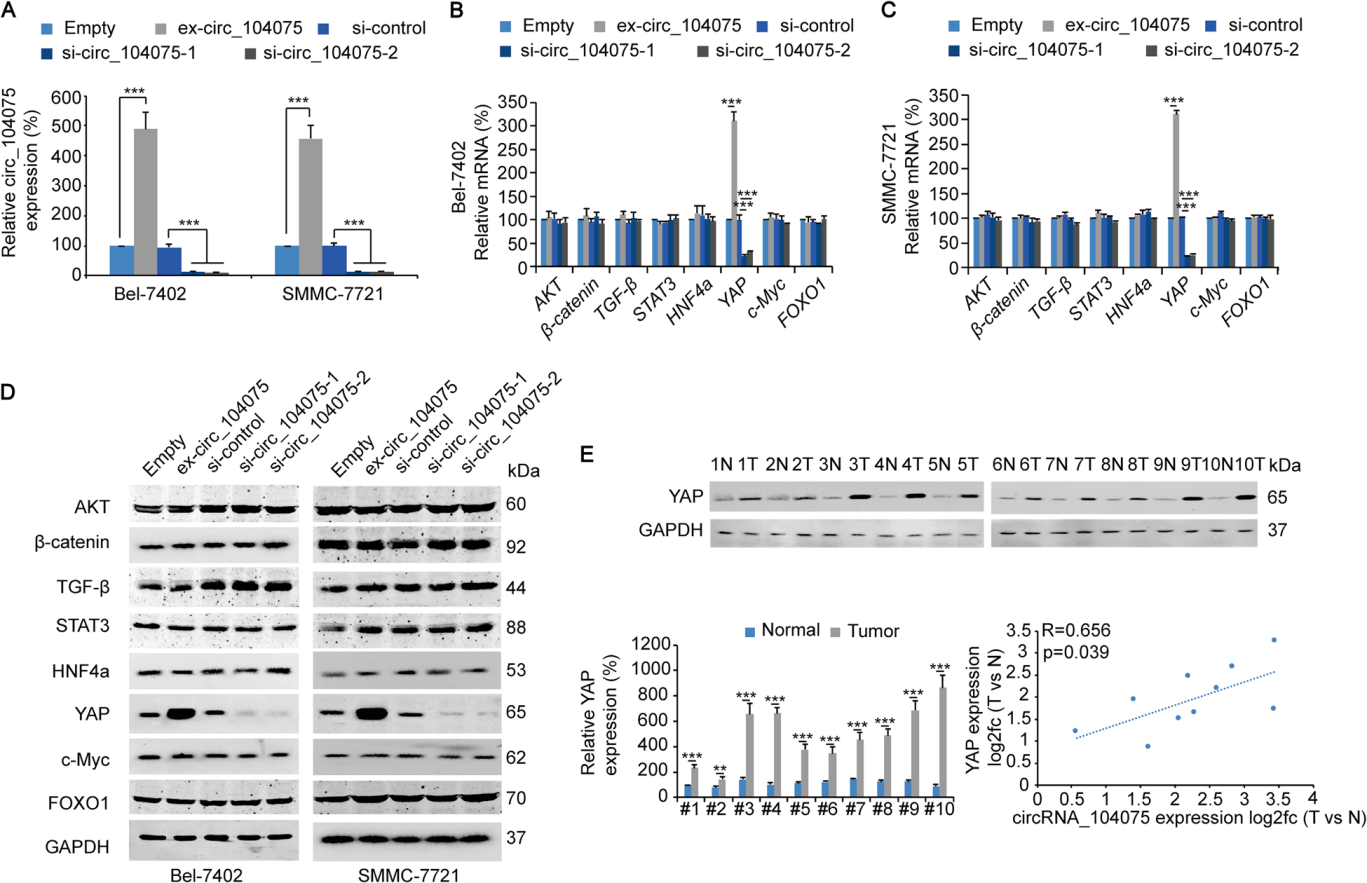
circ_104075 stimulated YAP expression. **a** circ_104075 was overexpressed or knocked down with the indicated plasmids, and circ_104075 expression was measured in Bel-7402 and SMMC-7721 cells using qPCR. **b**, **c** circ_104075 was overexpressed or knocked down with the indicated plasmids, and the mRNA level of the indicated HCC stimulators was measured in Bel-7402 (**b**) and SMMC-7721 (**c**) cells using qPCR. **d** circ_104075 was overexpressed or knocked down with the indicated plasmids, and the protein expressions of the indicated HCC stimulators were measured in Bel-7402 and SMMC-7721 cells using WB. **e** The YAP protein expression was detected using WB in ten pairs of HCC and adjacent normal tissues (upper), measured using the ImageJ software and normalized to the expression of GAPDH (lower left). Relationship between the YAP protein expression and circ_104075 expression was measured (lower right). The data are presented as the means + SD from three biological replicates. **p < 0.01, ***p < 0.001. The data shown in **a**–**c** and **e** (lower left) were analyzed using a one-way ANOVA test. The data shown in **e** (lower right) were analyzed using the Spearman rank-correlation analysis

Next, we investigated which tumor-promoting pathway was stimulated by circ_104075 in HCC. circ_104075 expression and siRNA vectors were designed (Fig. 4a) to test the expression of factors indicating the activity of HCC-promoting signaling pathways, including AKT^30^, β-catenin^31^, TGF-β^32^, STAT3^27^, HNF4a^25^, YAP^33^, c-Myc^28^, and FOXO1^29^. We observed that only the mRNA level of YAP was stimulated by circ_104075 overexpression and was suppressed by circ_104075 knockdown in both Bel-7402 (Fig. 4b) and SMMC-7721 cells (Fig. 4c). Similarly, the protein expression of YAP was promoted by circ_104075 overexpression and was inhibited by circ_104075 knockdown (Fig. 4d). YAP expression was higher in HCC tissues compared to those in normal tissues, and a positive correlation of the fold-change (HCC vs. normal) was observed between the circ_104075 mRNA level and YAP expression (*R* = 0.656, *p* = 0.039) (Fig. 4d). These data suggested the potential positive correlation between circ_104075 and YAP.

**Fig. 5:**
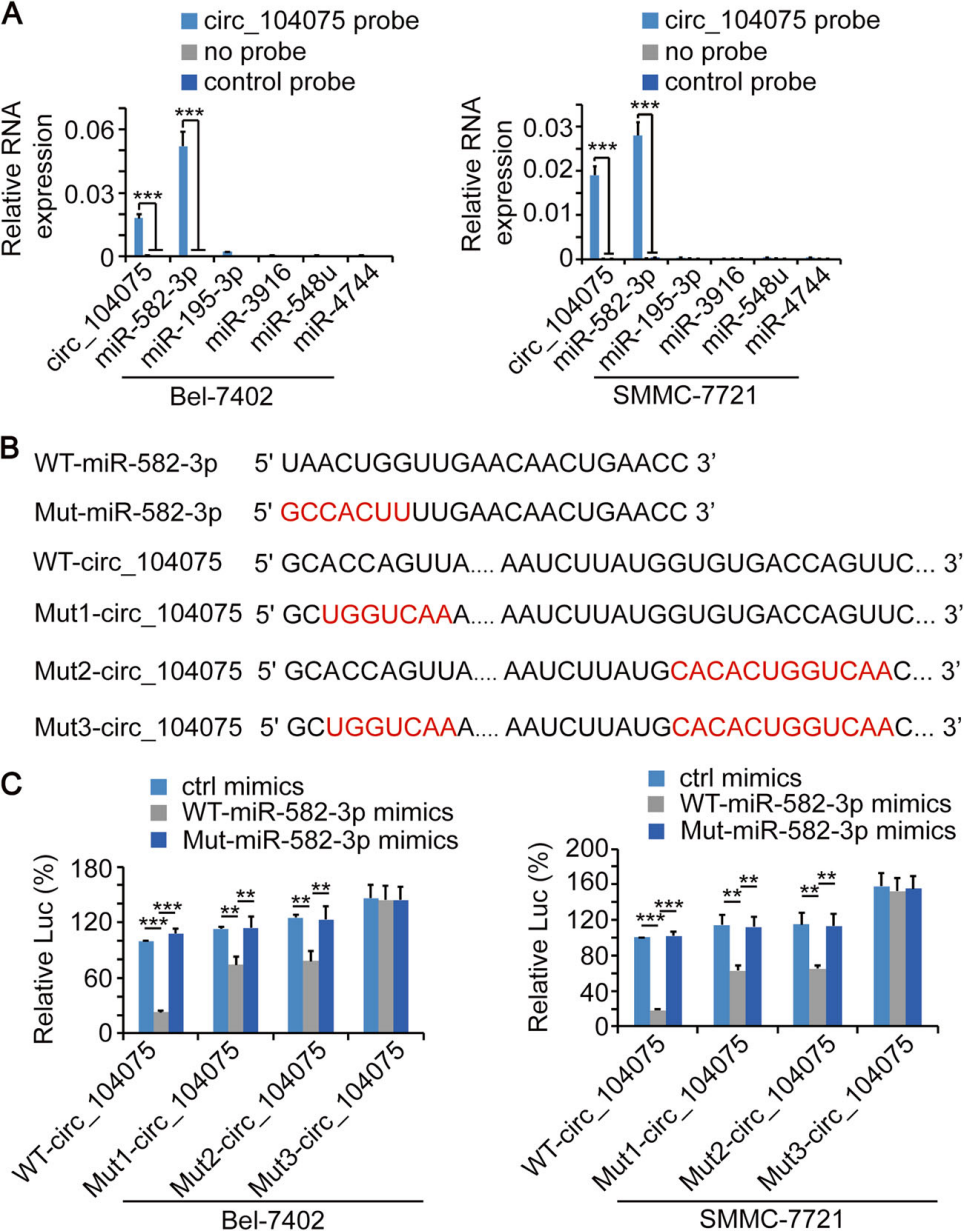
circ_104075 directly bound to miR-582-3p. **a** circ_104075 and indicated microRNA in Bel-7402 and SMMC-7721 cell lysis was pulled down and enriched with a circ_104075-specific probe and then detected by qPCR. **b** WT and mutant miR-582-3p and circ_104075 sequences were presented. Red fonts represented the mutant bases. **c** WT-miR-582-3p or Mut-miR-582-3p was overexpressed with the indicated plasmids. Activities of WT-circ_104075, Mut1-circ_104075, Mut2-circ_104075, or Mut3-circ_104075 luciferase reporter were measured using the dual-luciferase regent in Bel-7402 (left) and SMMC-7721 cells (right). The data are presented as the means + SD from three biological replicates. **p < 0.01, ***p < 0.001. The data shown in **a** and **c** were analyzed using a one-way ANOVA test

circRNA can function as a ceRNA to absorb microRNA and indirectly stimulate protein expression^34,35^. We predicted and screened the candidate microRNA targets of circ_104075 using the bioinformatics tool miRanda^36^. miR-582-3p, miR-195-3p, miR-3916, miR-548u and miR-4744 were predicted as the 5 most likely microRNAs to combine with circ_104075 with the highest total score (Supplementary Figure 1A). A circ_104075-specific probe (a segment of the biotin-tagged circ_104075 antisense oligonucleotides) was used to perform circRNA probe precipitation in circ_104075 overexpressing Bel-7402 a`nd SMMC-7721 cells to identify the microRNA that actually bound to circ_104075. The results indicated that miR-582-3p bound to circ_104075 in both Bel-7402 and SMMC-7721 cells (Fig. 5a).

To validate whether the predicted binding motifs in circ_104075 and miR-582-3p are responsible for their interaction, mutant miR-582-3p (Mut-miR-582-3p) mimics with mutated binding motifs and Mut1- (with the predicted binding motif mutated in the 5′end of circ_104075), Mut2- (with the predicted binding motif mutated near the 3′end of circ_104075) and Mut-3- (with both predicted binding motifs mutated in circ_104075) circ_104075 luciferase reporters were constructed (Fig. 5b). We observed that the miR-582-3p mimics significantly inhibited the luciferase activity of WT-circ_104075, Mut1-circ_104075, and Mut2-circ_104075 reporters, whereas the Mut-miR-582-3p mimics lost their ability to regulate the activity of circ_104075 reporters. Moreover, when both predicted binding motifs in circ_104075 were mutated, the luciferase activities of circ_104075 reporters were not regulated by miR-582-3p mimics (Fig. 5c), which suggested that both predicted binding motifs in circ_104075 and the other predicted binding motif in miR-582-3p played crucial roles in their interactions.

**Fig. 6:**
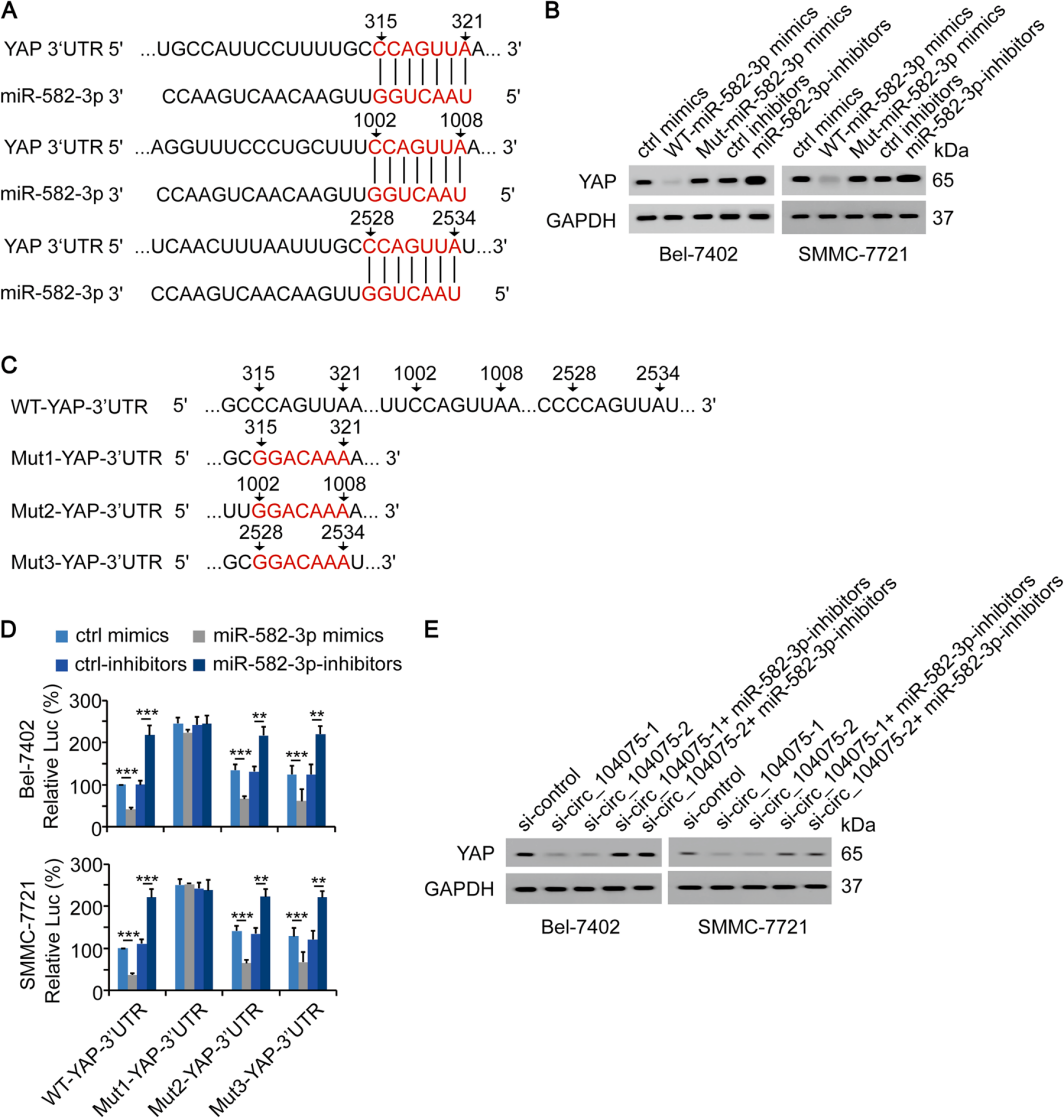
YAP-3′UTR was the target of miR-582-3p. **a** Sequence of complementary pairing bases (red fonts) in YAP 3′UTR and miR-582-3p was represented. **b** YAP expression was measured using WB via overexpression of WT-miR-582-3p or Mut-miR-582-3p or knockdown of miR-582-3p in Bel-7402 and SMMC-7721 cells. **c** WT and mutant YAP 3′UTR sequences were presented. Red fonts represented the mutant bases. **d** miR-582-3p was overexpressed or knocked down with indicated plasmids. Activities of WT-YAP-3′UTR, Mut1-YAP-3′UTR, Mut2-YAP-3′UTR, or Mut3-YAP-3′UTR were measured using the dual-luciferase regent in Bel-7402 (upper) and SMMC-7721 cells (lower). **e** si-circ_104075-1/2 with or without miR-582-3p inhibitors was transfected, and YAP expression was measured via WB in Bel-7402 and SMMC-7721 cells. The data are presented as the means + SD from three biological replicates. **p < 0.01, ***p < 0.001. The data shown in **d** were analyzed using a one-way ANOVA test

We further investigated whether miR-582-3p could directly bind to the 3′UTR region of the YAP mRNA. The TargetScan software was used to predict the potential binding motifs in miR-582-3p and the 3′UTR region of the YAP mRNA which are responsible for their interaction. The results indicated that three motifs in the 3′UTR region of YAP (315–321 region, 1002–1008 region and 2528–2534 region with the CCAGUUA motif) were possible binding motifs for the interaction with miR-582-3p (Fig. 6a). In miR-582-3p, the possible binding motif was in the 5′end (Fig. 6a), thus, we were able to use Mut-miR-582-3p mimics (used in Fig. 5c) to investigate this further. We observed that WT-miR-582-3p mimics negatively regulated the mRNA and protein expressions of YAP, whereas miR-582-3p-inhibitors exerted the opposite effects in both Bel-7402 and SMMC-7721 cell lines (Fig. 6b and Supplementary Figure 1B). However, Mut-miR-582-3p mimics lost the ability to downregulate the mRNA and protein expressions of YAP (Fig. 6b and Supplementary Figure 1B), demonstrating the vital role of the binding motif in miR-582-3p for its interaction with the 3′UTR region of YAP. Three potential binding motifs in the 3′UTR region of YAP were mutated (Fig. 6c), cloned into luciferase vectors and transfected into Bel-7402 and SMMC-7721 cells, respectively. We observed that only when the binding motif close to the 5′end (315-321 region in YAP 3′UTR) was mutated, the miR-582-3p mimics and miR-582-3p-inhibitors lost their abilities to regulate the activity of YAP 3′UTR. However, miR-582-3p still significantly regulated the luciferase activities of the other two reporters with mutated 1002–1008 or 2528–2534 region in YAP 3′UTR, suggesting that the 315–321 region in YAP 3′UTR might be the binding motif that was directly bound by miR-582-3p (Fig. 6d).

Compared to the control siRNA vector, si-circ_104075-1 and si-circ_104075-2 vectors significantly inhibited the mRNA and protein levels of YAP in both Bel-7402 and SMMC-7721 cells. However, when the miR-582-3p-inhibitors were transfected, the inhibition of YAP mRNA and protein levels were reversed, suggesting that miR-582-3p is essential in circ_104075-mediated regulation of YAP (Fig. 6e and Supplementary Figure 1C). Furthermore, miR-582-3p-inhibitors could reverse the cell viability and colony formation inhibition induced by si-circ_104075, and YAP knockdown could further inhibit the malignant phenotype stimulation induced by miR-582-3p-inhibitors (Supplementary Figure 1D–E). Collectively, these results demonstrated that circ_104075 functioned as a ceRNA to stimulate YAP dependent HCC tumorigenesis.

### The m^6^A modification at the 353~357 region of YAP 3′UTR induced the repression by miR-582-3p

**Fig. 7:**
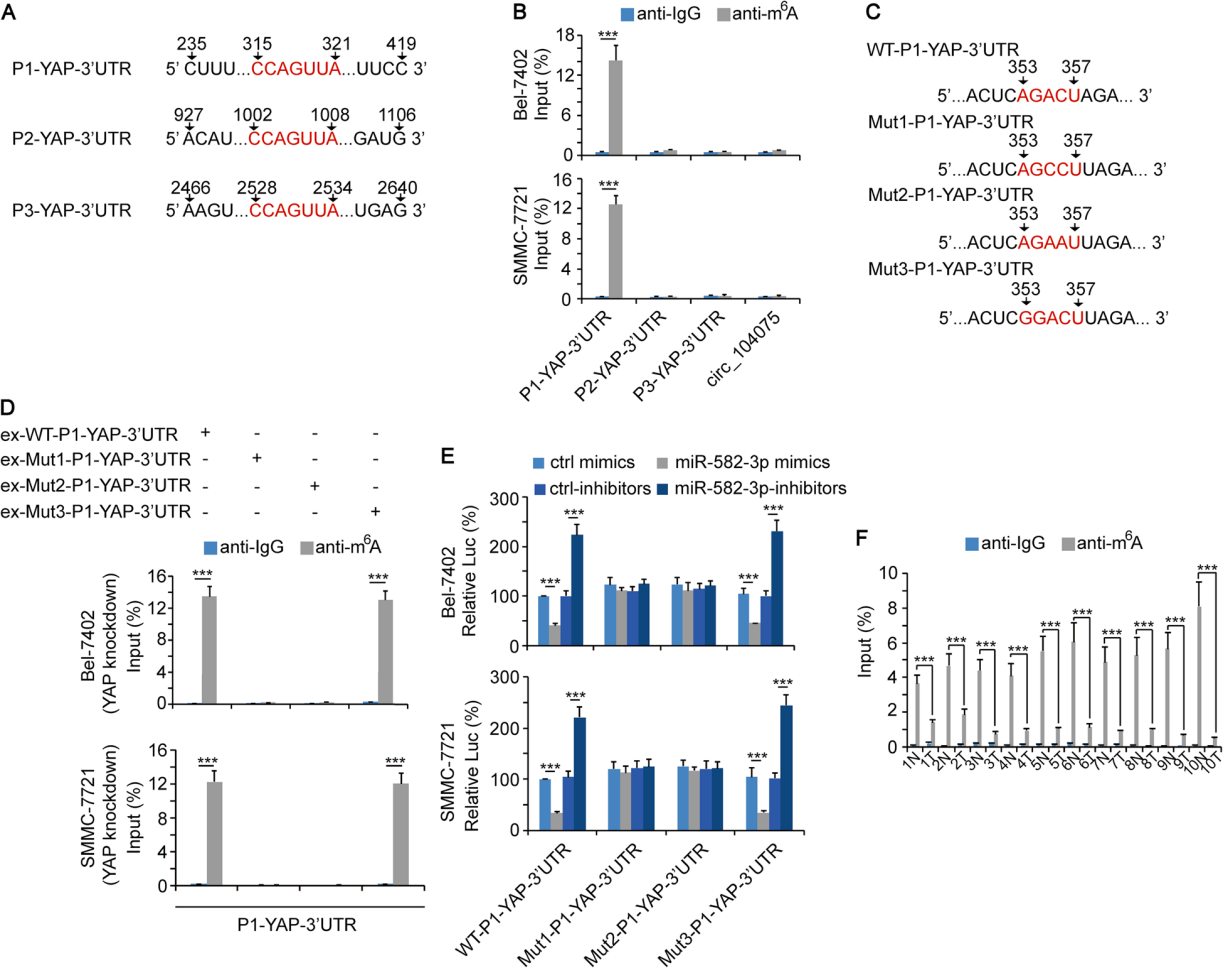
m^6^A modification in YAP-3′UTR promoted its interaction with miR-582-3p. **a** Sequences of YAP 3′UTR region that contained the miR-582-3p-binding motif. Red fonts represented the miR-582-3p binding motif. **b** The enrichment of m^6^A in Bel-7402 and SMMC-7721 cells at indicated regions of YAP 3′UTR was calculated as the percentage of the input RNA via RNA-IP using the m^6^A antibodies. A non-specific IgG was used as the negative control antibody. **c** WT or mutant m^6^A motifs in the P1 (235~ 419) regions of YAP 3′UTR. Red fonts represented the WT or mutant m^6^A motif. **d** WT-P1-YAP-3′UTR, Mut1-P1-YAP-3′UTR, Mut2-P1-YAP-3′UTR, or Mut-3-P1-YAP-3′UTR was overexpressed in YAP knockdown Bel-7402 (upper) or SMMC-7721 (lower) cells. The enrichment of m^6^A at P1 (235–419) regions of YAP 3′UTR was calculated as the percentage of the input RNA via RNA-IP using the m^6^A antibodies. A non-specific IgG was used as the negative control antibody. **e** miR-582-3p was overexpressed or knocked down using indicated plasmids. The activities of WT-P1-YAP-3′UTR, Mut1-P1-YAP-3′UTR, Mut2-P1-YAP-3′UTR, or Mut-3-P1-YAP-3′UTR were measured using the dual-luciferase regent in Bel-7402 (upper) and SMMC-7721 (lower) cells. **f** The enrichment of m^6^A in ten pairs of HCC and adjacent normal tissues at the P1 (235–419) regions of YAP 3′UTR was calculated as the percentage of the input RNA via RNA-IP using the corresponding antibodies. A non-specific IgG was used as the negative control antibody. The data are presented as the means + SD from three biological replicates. ***p < 0.001. The data shown in **b**, **d**, **e**, and **f** were analyzed using a one-way ANOVA test

m^6^A modification plays critical roles in regulating the stability of mRNA^37,38^. However, it remains unclear whether m^6^A modification could influence the interaction between microRNA and mRNA. Three pairs of qPCR primers were designed that contained three potential miR-582-3p-binding motifs in the 3′UTR of YAP, respectively (Fig. 7a). RNA-IP-qPCR was performed using anti-m^6^A to detect the level of m^6^A modification. Data showed that the m^6^A modification level of P1-YAP-3′UTR (235–419 region) was significantly upregulated. No significant m^6^A modification was detected in P2-YAP-3′UTR (927–1106 region and P3-YAP-3′UTR (2466–2640 region), as well as circ_104075 (Fig. 7b). We observed an AGACU motif in the 353–357 region of YAP 3′UTR, which is consistent with the RRACU (R represents for A or G) m^6^A motif (Fig. 7c). Next, we constructed expression vectors and luciferase reporters that harbored mutations of the AGACU m^6^A motif, in which the 355 adenine residue was replaced by cytosine (Mut1-P1-YAP-3′UTR), the 356 cytosine was replaced by adenine (Mut2-P1-YAP-3′UTR), or the 353 guanine was replaced by adenine (Mut3-P1-YAP-3′UTR), respectively (Fig. 7c). We overexpressed WT-P1-YAP-3′UTR, Mut1-P1-YAP-3′UTR, Mut2-P1-YAP-3′UTR, and Mut3-P1-YAP-3′UTR in YAP knockdown Bel-7402 and SMMC-7721 cells. RNA-IP-qPCR was performed to detect the m^6^A level of P1-YAP-3′UTR. Data showed that WT-P1-YAP-3′UTR and the Mut3-P1-YAP-3′UTR mutants exhibited significantly higher levels of m^6^A modification since a complete RRACU m^6^A motif was existed. However, Mut1- and Mut2-P1-YAP-3′UTR could not be m^6^A modified since their RRACU m^6^A motif was disrupted (Fig. 7d). Moreover, the luciferase activities of WT- and Mut3-P1-YAP-3′UTR reporters could be downregulated by miR-582-3p mimics and upregulated by miR-582-3p-inhibitors, whereas the luciferase activities of Mut1-P1-YAP-3′UTR and Mut2-P1-YAP-3′UTR reporters were not regulated by miR-582-3p. This result demonstrated that the m^6^A modification of the 353–357 region of YAP 3′UTR is essential for the interaction between YAP 3′UTR and miR-582-3p (Fig. 7e). We also evaluated the m^6^A modification levels of the 235–419 regions of YAP 3′UTR in HCC and adjacent normal tissues. Data showed that the m^6^A-modification levels were significantly higher in the normal tissues compared to those of the HCC tissues (Fig. 7f). These results suggested that the 353–357 region in YAP 3′UTR was m^6^A modified, and this modification was critical for miR-582-3p to exert its YAP inhibition function.

### Serum circ_104075 indicated the presence of liver cancer

**Fig. 8:**
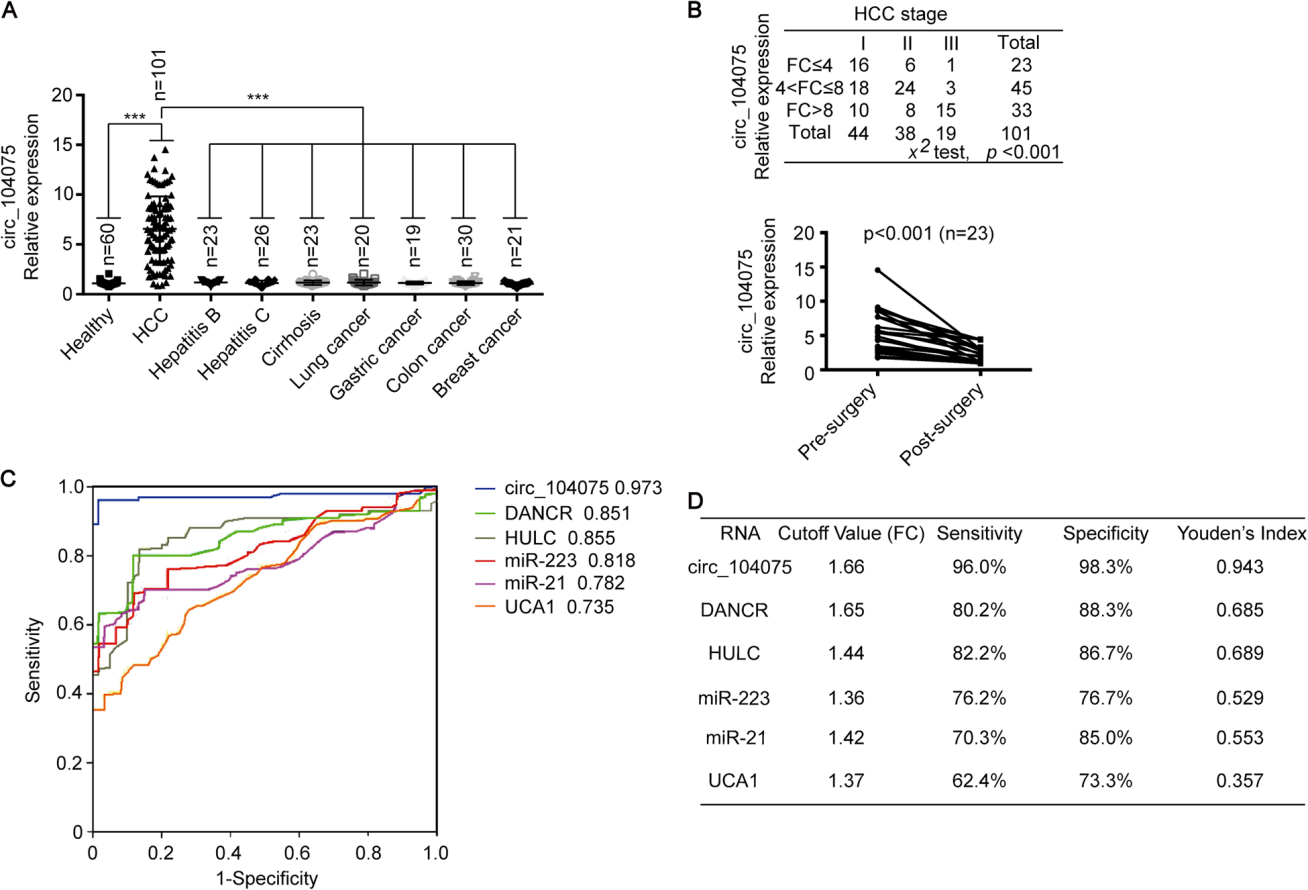
Serum circ_104075 is an HCC diagnostic biomarker. **a** Serum circ_104075 expression was measured in healthy individuals and patients with HCC, hepatitis B, hepatitis C, cirrhosis, lung cancer, gastric cancer, colon cancer and breast cancer using qPCR. **b** Association between the HCC stage and the circ_104075 expression fold change (compared to mean value of Healthy) was analyzed (upper). Pre-surgery and post-surgery differences in the serum circ_104075 levels were analyzed in HCC patients (lower). **c** ROC curves for serum circ_104075, DNACR, HULC, miR-223, miR-21 and UCA1 for the discrimination of patients with HCC from normal healthy individuals. **d** The cutoff value, sensitivity, specificity and Youden′s index for non-coding RNA biomarkers. The data are presented as the means + SD from three biological replicates. ***p < 0.001. The data shown in **a** were analyzed using a one-way ANOVA test. The data shown in (B, upper) were analyzed using a chi-square test. The data shown in **b** (lower) were analyzed using a two-way ANOVA test. FC, fold change

We measured serum circ_104075 levels in HCC patients, healthy individuals, and patients with other liver diseases (including hepatitis B, hepatitis C and cirrhosis), other cancers of the digestive system (including colon cancer and gastric cancer), breast cancer, and lung cancer. We found that the circ_104075 levels in HCC patients was higher than those in healthy individuals and patients with hepatitis B, hepatitis C, cirrhosis, lung cancer, gastric cancer, colon cancer and breast cancer; this result suggests that the elevated expression of circ_104075 was specific to HCC (Fig. 8a). Moreover, a higher stage of HCC was significantly associated with higher serum circ_104075 level (*p* = 0.000, Fig. 8b). Serum circ_104075 levels were analyzed before and after curative surgery, and we found that the serum circ_104075 level decreased significantly after surgery (Fig. 8b). We performed a receiver operating characteristic (ROC) curve analysis for circ_104075, DANCR, HULC, miR-223, miR-21, UCA1, AFP, DCP, and AFP-L3 according to the biomarker levels in HCC patients and healthy individuals. The area under the ROC curve (AUC-ROC) indicated that circ_104075 (AUC-ROC: 0.973) might be a better serum predictor for HCC compared to other non-coding RNA biomarkers (DANCR, AUC-ROC: 0.851; HULC, AUC-ROC: 0.855; miR-223, AUC-ROC: 0.818; miR-21, AUC-ROC: 0.782; UCA1, AUC-ROC: 0.735) (Fig. 8c) and classical protein biomarkers (AFP, AUC-ROC: 0.750; DCP, AUC-ROC: 0.771; AFP-L3: 0.766) (Supplementary Figure 2A). The best cutoff value of serum circ_104075 for predicting HCC was 1.66 (fold-change in HCC compared to healthy individuals), with a sensitivity of 96.0% and a specificity of 98.3%. The sensitivity and specificity of other RNA biomarkers (DANCR, HULC, miR-223, miR-21 and UCA1) (Fig. 8d) and classical protein biomarkers (AFP, DCP and AFP-L3) (Supplementary Figure 2B) for predicting HCC were lower than those of circ_104075, which suggests that circ_104075 is a promising serum biomarker for the diagnosis of HCC.

## Discussion

Here, we revealed that circ_104075 could absorb miR-582-3p to stimulate tumorigenesis via YAP. YAP is phosphorylated and suppressed by LATS, the kinase in the Hippo pathway^39^. When YAP is activated by other stimulations, such as O-GlcNAcylation, it loses its combination with LATS, translocates from the cytoplasm into the nucleus, and interacts with its dependent transcription factors, such as TEAD and RUNX1^40–42^. YAP is a well-accepted stimulator in HCC and is found to be upregulated in approximately 62% liver tissues of HCC patients^43,44^. Transgenic overexpression of YAP leads to the dysregulation of organ size and eventual HCC in mice^45^. We uncovered a novel circRNA-related pathway that stimulates YAP in HCC and provided new evidence that YAP promoted HCC occurrence and development.

We observed that circ_104075 activated YAP as a ceRNA to absorb the YAP inhibitor miR-582-3p. Numerous microRNA-binding sites exist on a wide variety of RNA transcripts; thus, all RNA transcripts that contain microRNA-binding sites can communicate with and regulate each other by competing for shared microRNAs, and act as ceRNAs^46^. mRNA, lncRNA and pseudogenes possess ceRNA activity^47^. In HCC, circRNA could function as ceRNA to stimulate or inhibit tumorigenesis. For instance, circMTO1 could act as a sponge of miR-9 and stimulate p21 expression to suppress HCC progression^22^. Another example is cSMARCA5, which could promote the expression of TIMP3, a well-known tumor suppressor, by sponging miR-17-3p and miR-181b-5p^35^. Here, we identified that circ_104075 acted as ceRNA to stimulate HCC development, which further demonstrate the critical role of circRNA as ceRNA in HCC.

m^6^A modification is the most abundant RNA modification in mammalian systems^48^. It plays diverse roles in the epigenetic regulation of RNA, including mRNA stability^37^, alternative splicing^49^, and microRNA biogenesis^50^. m^6^A modification could regulate the structure of methylated transcripts to prevent or induce the binding of other RNA. For instance, Yang et al. reported that m^6^A modification of lincRNA1281 could ensure its interaction with sequestering the pluripotency-related let-7 family and maintain the differentiation ability of mESC^51^. In this study, we observed that m^6^A modification in the YAP 3′UTR induced the interaction of miR-382-5p and led to the inhibition of YAP. This finding provided new evidence that m^6^A modification of RNA transcripts could impact its stability via regulating its interaction with microRNA.

It is interesting that m^6^A modification at 353–357 region induced miR-582-3p binding to 315–321 region in the YAP 3′UTR. Human antigen R (HuR), an RNA stabilizer protein that increases RNA stability by blocking microRNA targeting, tends to bind to the 3′-UTR of thousands of transcripts^52^. Wang et al. reported that m^6^A methylation prevents HuR binding to target RNA, whereas it induces microRNA binding^53^. We speculated that m^6^A modification at 353–357 region might inhibit HuR binding, and subsequently induce miR-582-3p binding to the 315–321 region in the YAP 3′UTR. Previous studies have also reported that m^6^A modification could enhance the accessibility of RNA binding motifs within 50 nucleotides to induce binding between the RNA transcript and protein by altering the structure of the RNA transcript^54^. Therefore, another probable reason why m^6^A modification promoted the binding between YAP 3′UTR and microRNA-582-3p is that m^6^A modification might alter the spatial structure of YAP 3′UTR. Additionally, microRNA modulates m^6^A levels by targeting the m^6^A reader YTH domain family 2 or writer methyltransferase-like 3^53,55^. Whether miR-582-3p regulates m^6^A levels of YAP 3′UTR or global m^6^A levels needs to be further investigated.

Non-coding RNA-based diagnosis might be a new choice in clinical practice since it has remarkably high specificity compared to protein-based diagnosis^6^. Compared to other non-coding RNAs, circRNA is not easily degraded by the exonuclease RNase R because of its covalently closed loop structure, and it can stably exist in body fluids^56^. The diagnostic performance of circRNA has been reported in gastric^57^, laryngeal^58^, and lung cancers^59^, and AML^60^. We observed that circ_104075 exhibits a sensitivity of 96.0% and a specificity of 98.3% in HCC diagnosis, which is better than that of other non-coding RNA biomarkers, such as DANCR, HULC, miR-223, miR-21, and UCA1. However, serum circ_104075 measurements have not been standardized, and the cutoff value of circ_104075 in this study may not be suitable for other studies. Therefore, the large-scale studies and multicenter-trials need to be performed to validate the diagnostic performance of serum circ_104075.

In summary, our study elucidates how circ_104075 is upregulated in HCC and its downstream mechanism to stimulate HCC tumorigenesis. circ_104075 might serve as a novel diagnostic biomarker and a therapeutic target in HCC.

## Materials and methods

### Serum and tissue samples

Serum samples were collected from patients who were diagnosed with primary HCC (mean age ± SD, 64.59 ± 5.71 years; male: female ratio, 1.66:1), hepatitis B (mean age ± SD, 39.75 ± 5.52 years; male: female ratio, 1.3:1), hepatitis C (mean age ± SD, 57.48 ± 10.21 years; male: female ratio, 1.17:1), cirrhosis (mean age ± SD, 57.42 ± 8.75 years; male: female ratio, 2.29:1), colon cancer (mean age ± SD, 62.18 ± 10.44 years; male: female ratio, 3.29:1), gastric cancer (mean age ± SD, 61.25 ± 13.02 years; male: female ratio, 2.8:1), lung cancer (mean age ± SD, 54.43 ± 11.01 years; male: female ratio, 3:1) and breast cancer (mean age ± SD, 39.73 ± 10.67 years; all female patients) at Shanghai Ruijin Hospital (Shanghai, China) between May 2015 and May 2017 for this study. A total of 60 healthy individuals (mean age ± SD, 51.61 ± 13.88 years; male: female ratio, 1.22:1) were recruited between May 2015 and May 2017 at Shanghai Ruijin Hospital as control subjects. Blood samples were collected before or after curative surgery, and the serum was immediately centrifuged, aliquoted and stored at −80 °C. Ten pairs of tumor and adjacent liver tissues were acquired at Shanghai Ruijin Hospital between May 2015 and May 2017. The diagnoses of HCC, colon cancer, gastric cancer, breast cancer and lung cancer were confirmed via histopathological analyses. Hepatitis B or C patients were confirmed through detection of more than 1 × 10^3^ copies of HBV or HCV nucleic acids in the serum (Kehua, Shanghai, China). The study protocol was conducted in accordance with the ethical guidelines of the 1975 Declaration of Helsinki. Samples were collected under institutional approvals. Informed written consent was obtained from all patients.

### Mouse experiments

*HNF4a* knockout (*HNF4a-/-*) mice were generated using CRISPR/Cas9 in C57BL/6 mice and purchased from the Shanghai Research Center of the Southern Model Organisms (Shanghai, China). For *HNF4a-/-* mice, the sgRNAs used to knock out *HNF4a* were gRNA1 (5′-CTTATATCCTTCCGCTCTCTGGG-3′) and gRNA2 (5′- AACGAGGATACCACGTCAGAAGG-3′). HNF4a-/- mice were genotyped using primers F1 (5′- GATGGCTTCTGGAGATGGTGGTA-3′) and F2 (5′- CAAGGCAAGGTTATGGGAGTGTGG-3′), giving rise to 1 band (229 bp) in *HNF4a-/-* mice, 2 bands (229 and 1082 bp) in heterozygous mice and 1 band (1082 bp) in WT mice. PCR was performed to identify *HNF4a-/-*, heterozygous and WT mice (Supplementary Figure 3). All mouse experiments were performed in accordance with the institutional guidelines of the Shanghai Tenth People′s Hospital, which has permission for animal experimentation from the Science and Technology Commission of the Shanghai Municipality.

### Cell culture, vectors, and primers

The liver cancer cell lines Bel-7402 (Cell bank of Chinese Academy of Sciences, Shanghai, China), SMMC-7721 (Cell bank of Chinese Academy of Sciences, Shanghai, China), Huh7 (Cobioer, Nanjing, China), HepG2 (Cobioer, Nanjing, China), Hep1 (Cell bank of Chinese Academy of Sciences, Shanghai, China), and Bel-7404 (Cell bank of Chinese Academy of Sciences, Shanghai, China) and the hepatocyte lines THLE-3 (Biovector, NTCC, Beijing, China) and HL-7702 (Cell bank of Chinese Academy of Sciences, Shanghai, China) were cultured in DMEM. TEAD, CREB, TFCP2, STAT3, c-Myc, FOXO1, TEAD-sh1, CREB-sh1, TFCP2-sh1, STAT3-sh1, c-Myc-sh1, and FOXO1-sh1 plasmids were acquired from previous studies^40,42,61–66^. HNF4a plasmids were purchased from Origene (Beijing, China). HNF4a-sh1 plasmids were purchased from Open Biosystems (Huntsville, AL, USA). The ex-circ_104075, si-circ_104075-1/2, HNF4a-sh2, YAP-sh (targeting 3′UTR), WT-miR-582-3p mimics, Mut-582-3p-mimics, miR-582-3p-inhibitors, ex-WT-P1-YAP-3′UTR, ex-Mut1-P1-YAP-3′UTR, ex-Mut2-P1-YAP-3′UTR, ex-Mut3-YAP-3′UTR plasmids and WT-circ_104075, Mut1-circ_104075, Mut2-circ_104075, and Mut3-circ_104075 luciferase reporters were purchased from Biolink (Shanghai, China). Regions of the circRNA_104075 promoter or YAP 3′UTR were amplified via PCR from the gDNA of Bel-7402 cells and cloned into pGL4.21 (Promega, Madison, WI, USA) vectors. Mutant promoter plasmids were constructed using overlapping PCR. The primers are listed in Supplementary Table 1.

### Western blotting (WB)

Cells were harvested, washed in cold PBS, and lysed in Western/IP lysis buffer (Beyotime, Shanghai, China). The whole-cell lysates were centrifuged at 12,000 rpm for 15 min at 4 °C. Protein concentrations were measured using the Bradford method. Proteins were separated via SDS-PAGE, and transferred onto a nitrocellulose membrane. The membranes were blocked using 5% evaporated milk and 1% Tween-20 in PBS for 1 h and were incubated with primary antibodies dissolved in PBS containing 1% Tween-20 overnight at 4 °C. The primary antibodies were anti-YAP (Abcam, Hong Kong, China, #ab52771), anti-β-catenin (Abcam, #ab32572), anti-TGF-β (Abcam, #ab31013), anti-STAT3 (Abcam, #ab68153), anti-HNF4a (Abcam, #ab41898), anti-c-Myc (Abcam, #ab32072), anti-FOXO1 (Abcam, #ab39670) and anti-GAPDH (Cell Signaling Technology (CST), Boston, MA, USA, #5174). On the second day, the blots were incubated with the appropriate secondary antibody: IRDye 800CW goat anti-mouse IgG (LICOR, Lincoln, NE, USA, #926-32210), IRDye 800CW goat anti-rabbit IgG (LICOR, #926-32211), anti-rabbit IgG, HRP-linked antibody (CST, #7074) or anti-mouse IgG, HRP-linked antibody (CST, #7076). The signals were detected using the Odyssey two-color infrared laser imaging system (LICOR) or HRP-based chemiluminescence analysis.

### Real-time quantitative PCR (qPCR)

Total RNAs were extracted using Trizol (Ambion, Carlsbad, CA, USA) and were detected using a Nanodrop 1000 spectrophotometer (Thermo Scientific, Waltham, MA, USA); one microgram of total RNA was reverse-transcribed into complementary DNA using PrimeScript™ RT reagent Kit (Perfect Real Time) (TaKaRa, Dalian, China). Quantitative PCR was performed using the SYBR premix Ex Taq (TaKaRa) kit in the Real-Time PCR System (Applied Biosystems ABI 7500). The primers are listed in Supplementary Table 1.

### Dual-luciferase analysis

Luciferase reporter vectors were cotransfected into liver cancer cells with a Renila luciferase expression plasmids, grown in 24-well plates for 48 h, and harvested for passive lysis (Promega, Madison, WI, USA). Luciferase activities were measured using dual-luciferase reagent (Promega).

### Chromatin immunoprecipitation (ChIP)

ChIP was performed using the kits from Active Motif (Carlsbad, CA, USA). Cells (2 × 10^7^) were fixed using 1% formaldehyde, washed with PBS and lysed in lysis buffer. After sonication, the protein-DNA complexes were incubated overnight with antibody-coupled protein G beads at 4 °C. DNA was eluted in 1% SDS/0.1 M NaHCO_3_, reversed cross-link at 65 °C, and purified via phenol/chloroform extraction and ethanol precipitation. The samples obtained were subjected to qPCR analysis. The antibodies used were anti-HNF4a (Abcam, #ab41898) and anti-IgG (CST, #3900).

### RNA-IP assay

RNA-IP was performed using the kits from Active Motif, and immunoprecipitation was performed using the control IgG (CST, #3900) or anti-m^6^A antibody (Abcam, ab151230). Next, the RNA complexes were isolated through phenol-chloroform extraction and analyzed via qPCR assays.

### Cell viability and colony formation assay

For the methyl thiazol tetrazolium (MTT)-based cell-proliferation assay, Bel-7402 and SMMC- 7721 cells (3000 cells per well) were seeded in a 96-well plate, treated with 5 mg/ml MTT. Five days later, and lysed in DMSO after 4 h. Absorbance was measured at 595 nm. For soft-agar colony formation assay, Bel-7402 and SMMC-7721 cells (6000 cells per well) were seeded a 6-well plate of 0.3% agarose in DMEM media containing 10% FBS. Colonies from 12 fields of view were counted 2 weeks later.

### circRNA probe precipitation

Biotin-labeled circ_104075 probe which (5′-TCATTTGTCTTCTAACTGGTGCAGAATTCAGAGGAGAAGAAACA-3′-biotin) was synthesized by Sangon Biotech (Shanghai, China). circ_104075 was overexpressed in Bel-7402 and SMMC-7721 cells, respectively, because overexpression of circ_104075 enables the circ_104075 probe to bind more circ_104075, and it is helpful for identifying the microRNAs that specifically bind to circ_104075. The cells were fixed using 4% formaldehyde for 10 min, lysed, sonicated, and centrifuged. Next, 50 μl of the supernatant was extracted as input, and the remaining amount was incubated with circ_104075 probe and Dynabeads M-280 Streptavidin (Thermo Scientific) overnight at 30 °C. The next day, the mixture was washed and incubate with 200 μl of lysis buffer and proteinase K to reverse the cross-linking. Finally, the RNA mixture was extracted using Trizol and was detected via qPCR^22,67^.

### Enzyme linked immunosorbent assay (ELISA)

The concentrations of AFP, DCP, and AFP-L3 were detected using ELISA. Samples were diluted (1:4) in dilution buffer, and added to 96-well microtiter plates for analyses. ELISA kits were purchased from Lichen Biotech Ltd (Shanghai, China). ELISA experiments were performed in strict accordance with the manufacturers’ guidelines. Signals were detected by measuring the absorbance at 450 nm in a microplate reader.

### Statistical analysis

Tests used to examine the differences between groups included one-way and two-way ANOVA, the chi-square test and the Spearman rank-correlation analysis. *p* < 0.05 was considered statistically significant.

## Electronic supplementary material

Supplementary Figure 1

Supplementary Figure 2

Supplementary Figure 3

Supplementary Table 1

Supplementary Figure Legends
