# Supplementary figures and images for: circRNA_104075 stimulates YAP-dependent tumorigenesis through the regulation of HNF4a and may serve as a diagnostic marker in hepatocellular carcinoma
Source: Cell Death Dis. 2018 Oct 25;9(11):1091. doi: 10.1038/s41419-018-1132-6 (PMC6202383; doi:10.1038/s41419-018-1132-6)

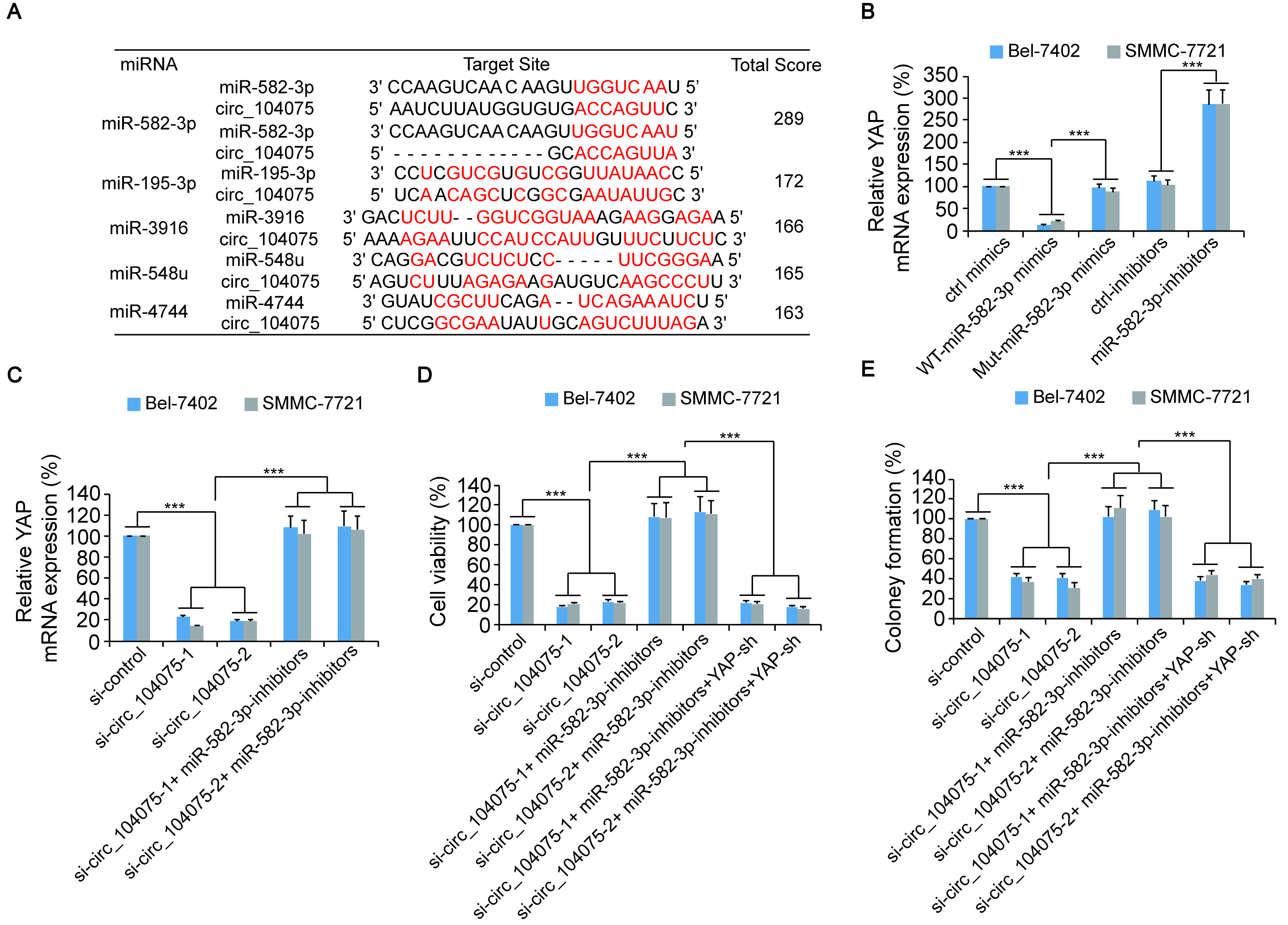

Supplement: Supplementary file 1 — Supplementary Figure 1 [file 41419_2018_1132_MOESM1_ESM.tif]

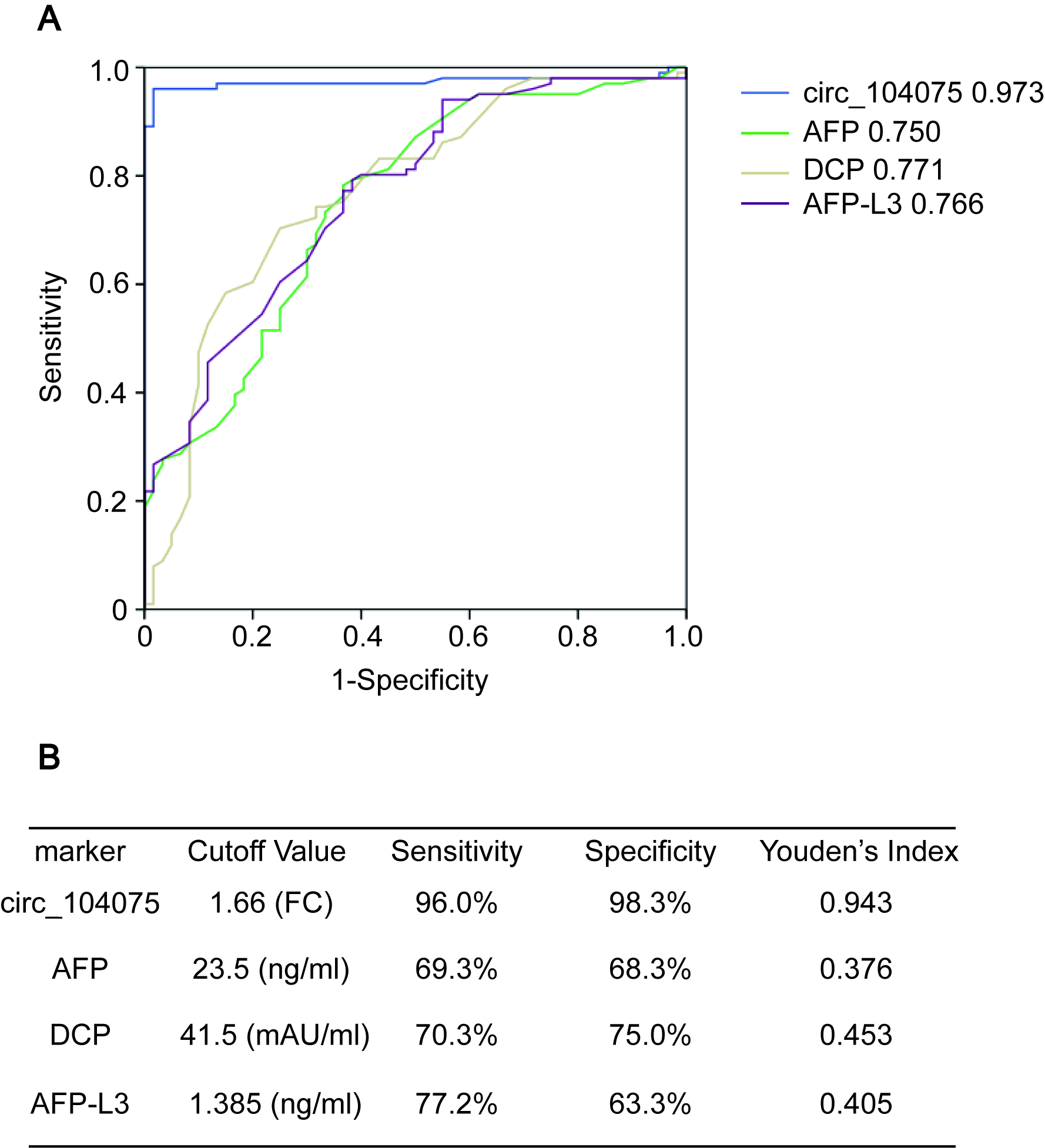

Supplement: Supplementary file 2 — Supplementary Figure 2 [file 41419_2018_1132_MOESM2_ESM.tif]

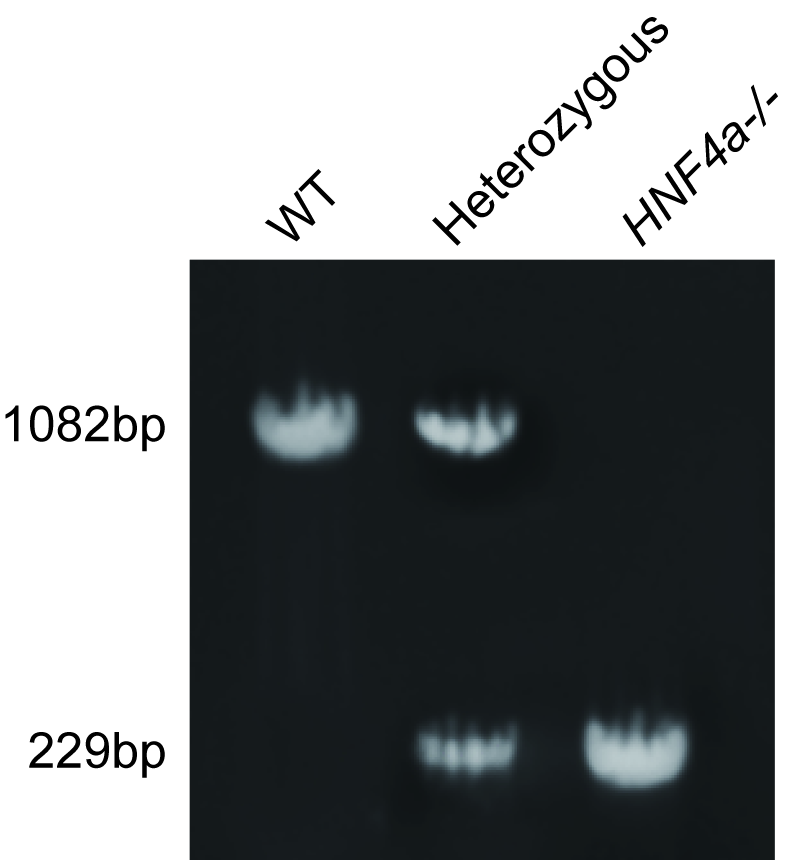

Supplement: Supplementary file 3 — Supplementary Figure 3 [file 41419_2018_1132_MOESM3_ESM.tif]
