# Supplementary material for: circRNA_104075 stimulates YAP-dependent tumorigenesis through the regulation of HNF4a and may serve as a diagnostic marker in hepatocellular carcinoma
Source: Cell Death Dis. 2018 Oct 25;9(11):1091. doi: 10.1038/s41419-018-1132-6 (PMC6202383; doi:10.1038/s41419-018-1132-6)
Supplement: Supplementary file 4 — Supplementary Table 1 [file 41419_2018_1132_MOESM4_ESM.docx]

**Primers used for qPCR**

| F-qPCR-circ_104075 | GAAGATGTCAAGCCCTTTAGC |
| --- | --- |
| R-qPCR-circ_104075 | GAGTTGCTTAGCTTTCATTTGTC |
| F-qPCR-DANCR | AGCTCCAGGAGTTCGTCTCTTAC |
| R-qPCR-DANCR | CCATTTCTTTACCAGCAACAGGA |
| F-qPCR-HULC | GCATCAAGCAAGAAGTTTCCTG |
| R-qPCR-HULC | AATTTGCCACAGGTTGAACACT |
| F-qPCR-UCA1 | ATCTCCTCGGCTTAGTGGCTG |
| R-qPCR-UCA1 | TGGTGTGCTATAAATGACCGGGT |
| F-qPCR-miR-21 | TAGCTTATCAGACTGATGTTGA |
| RT-qPCR-miR-21 | GTCGTATCCAGTGCAGGGTCCGAGGTATTCGCACTGGATACGACTCAACA |
| F-qPCR-miR-223 | CGTGTATTTGACAAGCTGAGTT |
| RT-qPCR-miR-223 | GTCGTATCCAGTGCAGGGTCCGAGGTATTCGCACTGGATACGACAACTCA |
| F-qPCR-AKT | ATGAACGAGTTTGAGTACCTGAAG |
| R-qPCR-AKT | TCCTTGGCCACGATGACTTCCT |
| F-qPCR-β-catenin | GGTCACCTGTGCAGCTGGAATTC |
| R-qPCR-β-catenin | AACTGCATTCTGGGCCATCTCT |
| F-qPCR-TGF-β | ATACCTCAGCAACCGGCTGCTG |
| R-qPCR-TGF-β | TGTGTTATCCCTGCTGTCACA |
| F-qPCR-STAT3 | CATCATGGGCTTTATCAGTAAG |
| R-qPCR-STAT3 | ACCAGTGGAGACACCAGGATAT |
| F-qPCR-HNF4a | TTGCCAGCATCGCAGATGTGTG |
| R-qPCR-HNF4a | AGCACGTCCTTGAACACCATGGA |
| F-qPCR-YAP | ATGCGGAATATCAATCCCAGCAC |
| R-qPCR-YAP | TCCACTGTCTGTACTCTCATCT |
| F-qPCR-c-Myc | AAGACTCCAGCGCCTTCTCTC |
| R-qPCR-c-Myc | AGCAGAAGGTGATCCAGACTCT |
| F-qPCR-FOXO1 | AAATGCCAGCTTTGTACAGGTC |
| R-qPCR-FOXO1 | CAACCAGGGCCTGAAACGTTGA |
| F-qPCR-circ_104075-promoter (-1482~-1296) | GAGACACGGTTTCACCATGTTGGCCAG |
| R-qPCR-circ_104075-promoter (-1482~-1296) | CCAGCATTTGATGATGGAC |
| F-qPCR-circ_104075-promoter (-1655~-1483) | CCAAGACGGAGTCTTGCTC |
| R-qPCR-circ_104075-promoter (-1655~-1483) | GCTACTAAAAATACACAC |
| F-qPCR-circ_104075-promoter (-1295~-1104) | TTGGGGGTAGAAATTTTGC |
| R-qPCR-circ_104075-promoter (-1295~-1104) | GCAATTTTTAGAGAAAAGCAG |
| F-qPCR-circ_104075-promoter (-1103~-897) | GCGGAAGAAATGATGTTTAC |
| R-qPCR-circ_104075-promoter (-1103~-897) | TTATAGGAGGCTGAGGCAGGAG |
| F-qPCR-circ_104075-promoter (-896~-690) | GAGTAGCTGGGACTACA |
| R-qPCR-circ_104075-promoter (-896~-690) | TTTCACTTGTGTATGCAG |
| F-qPCR-circ_104075-promoter (-689~-483) | GATTGAGGATATTTATGG |
| R-qPCR-circ_104075-promoter (-689~-483) | CTCTGAAGGCTGAGGCAGGAAG |
| F-qPCR-circ_104075-promoter (-482~-307) | TAGCTGGGGACTAC |
| R-qPCR-circ_104075-promoter (-482~-307) | TATTCACGAACAGTTCCCCG |
| F-qPCR-circ_104075-promoter (-306~-157) | CTGTCCTCTGCGGGAGATC |
| R-qPCR-circ_104075-promoter (-306~-157) | TTGCGAGCAGGAGCGGAGAGAG |
| F-qPCR-circ_104075-promoter (-156~-1) | CCTCGCGGTTCCTG |
| R-qPCR-circ_104075-promoter (-156~-1) | GGCGGAGCCTCCGCCGCTTCCC |
| F-qPCR-miR-582-3p | TAACTGGTTGAACAACTGAACC |
| RT-qPCR-miR-582-3p | GTCGTATCCAGTGCAGGGTCCGAGGTATTCGCACTGGATACGACGGTTCA |
| F-qPCR-miR-195-3p | CCAATATTGGCTGTGCTGCTCC |
| RT-qPCR-miR-195-3p | GTCGTATCCAGTGCAGGGTCCGAGGTATTCGCACTGGATACGACGGAGCA |
| F-qPCR-miR-3916 | AAGAGGAAGAAATGGCTGGTTCTCAG |
| RT-qPCR-miR-3916 | GTCGTATCCAGTGCAGGGTCCGAGGTATTCGCACTGGATACGACCTGAGA |
| F-qPCR-miR-548u | CAAAGACTGCAATTACTTTTGCG |
| RT-qPCR-miR-548u | GTCGTATCCAGTGCAGGGTCCGAGGTATTCGCACTGGATACGACCGCAAA |
| F-qPCR-miR-4744 | TCTAAAGACTAGACTTCGCTATG |
| RT-qPCR-miR-4744 | GTCGTATCCAGTGCAGGGTCCGAGGTATTCGCACTGGATACGACCATAGC |
| F-qPCR-P1-YAP-3'UTR | GCCTTTTGCTATTAAAACTACTG |
| R-qPCR-P1-YAP-3'UTR | GGAAGCTGAAGAAACAACTGTTG |
| F-qPCR-P2-YAP-3'UTR | ACATACACACACCCAAACATAAC |
| R-qPCR-P2-YAP-3'UTR | CATCTGCAGACTGCCCCAACCAG |
| F-qPCR-P3-YAP-3'UTR | AAGTACACCCACAAAACAATATG |
| R-qPCR-P3-YAP-3'UTR | CTCAAAAAGGGAAAGGACTCTTAGGTCTC |
| F-qPCR-GAPDH | ATCATCCCTGCCTCTACTGG |
| R-qPCR-GAPDH | GTCAGGTCCACCACTGACAC |

**Primers used for construction of luciferase reporters**

| F-WT-luc-circ_104075 promoter | CTAGGGTACCTGCAGAACCTGAGTATACCAAAAG |
| --- | --- |
| R-WT-luc-circ_104075 promoter | GTACGCTAGCGGCGGAGCCTCCGCCGCTTC |
| F-Mut-luc-circ_104075 promoter | GGGATTACAGGCGTGAGCCACCGCGC |
| R-Mut-luc-circ_104075 promoter | TCACGCCTGTAATCCCGGAGGCTGAGGCGGGTGGATCACAA |
| F-WT-luc-YAP-3'UTR | CTAGGGTACCTCAGGCAGACTGAATTCTAAATC |
| R-WT-luc-YAP-3'UTR | CGGCGGAGCTCTTTTTTTTTTAAGAG |
| F-Mut1-luc-YAP-3'UTR | AATGTTCACCAATCATTTTAAC |
| R-Mut1-luc-YAP-3'UTR | atgattggtgaacattTTTGTCCgcaaaaggaatggcatcca |
| F-Mut2-luc-YAP-3'UTR | ATTTTTAAAATGGTAGCGCTTTGTATGC |
| R-Mut2-luc-YAP-3'UTR | ctaccattttaaaaatTTTGTCCaaagcagggaaacctaa |
| F-Mut3-luc-YAP-3'UTR | TACCTCAGTGTTGTAGCAGTACTG |
| R-Mut3-luc-YAP-3'UTR | aacactgaggtaTTTGTCCgcaaattaaagttgaggggta |
| F-WT-luc-P1-YAP-3'UTR | CTAGGGTACCGCCTTTTGCTATTAAAACTACTG |
| R-WT-luc-P1-YAP-3'UTR | CGTAGGAGCTGGAAGCTGAAGAAACAACTGTTG |
| F-Mut-luc-P1-YAP-3'UTR | TAGAAGTCAGATGCTTCATGTCACA |
| R-Mut1-luc-P1-YAP-3'UTR | gaagcatctgacttctaagGctgagtatttagttaaaatg |
| R-Mut2-luc-P1-YAP-3'UTR | gaagcatctgacttctaaTTctgagtatttagttaaaatg |
| R-Mut3-luc-P1-YAP-3'UTR | gaagcatctgacttctaagTCCgagtatttagttaaaatg |
