# Supplementary material for: circRNA_104075 stimulates YAP-dependent tumorigenesis through the regulation of HNF4a and may serve as a diagnostic marker in hepatocellular carcinoma
Source: Cell Death Dis. 2018 Oct 25;9(11):1091. doi: 10.1038/s41419-018-1132-6 (PMC6202383; doi:10.1038/s41419-018-1132-6)
Supplement: Supplementary file 5 — Supplementary Figure Legends [file 41419_2018_1132_MOESM5_ESM.docx]

**Supplementary Figure 1. circ_104075 stimulated liver tumorigenesis via YAP by absorbing miR-582-3p.**

(A) Top 5 predicted microRNAs that bound to circ_104075 are displayed. Red fonts represented the complementary pairing bases.

(B) YAP mRNA level was measured using qPCR with WT- or Mut-miR-582-3p overexpression or miR-582-3p knockdown in Bel-7402 and SMMC-7721 cells.

(C) si-circ_104075-1/2 was transfected with or without miR-582-3p inhibitors, and the YAP mRNA level was measured via qPCR in Bel-7402 and SMMC-7721 cells.

(D-E) Cell viability (D) and colony formation (E) was measured with indicated plasmids transfected in Bel-7402 and SMMC-7721 cells.

The data are presented as the means + SD from three biological replicates. ***p<0.001. The data shown in (B), (C), (D) and (E) were analyzed using a one-way ANOVA test.

**Supplementary Figure 2. Diagnostic value of circ_104075 is better than classical biomarkers**

(A) ROC curves for serum circ_104075, AFP, DCP and AFP-L3 for the discrimination of patients with HCC from normal healthy individuals.

(B) The cutoff value, sensitivity, specificity and Youden’s index for circ_104075, AFP, DCP and AFP-L3.

**Supplementary Figure 3. Identification for *HNF4a* knockout mice**

*HNF4a-/-*, heterozygous and WT mice were identified by PCR experiments.
